# Identification and functional analysis of two *serotonin N-acetyltransferase* genes in maize and their transcriptional response to abiotic stresses

**DOI:** 10.3389/fpls.2024.1478200

**Published:** 2024-10-01

**Authors:** Xiaohao Guo, Le Ran, Xinyu Huang, Xiuchen Wang, Jiantang Zhu, Yuanyuan Tan, Qingyao Shu

**Affiliations:** ^1^ State Key Laboratory of Rice Biology and Breeding, and Zhejiang Provincial Key Laboratory of Crop Germplasm Innovation and Exploitation, The Advanced Seed Institute, Zhejiang University, Hangzhou, China; ^2^ School of Biological Science and Technology, University of Jinan, Jinan, China; ^3^ Zhejiang University – Wuxi Xishan Joint Modern Agricultural Research Centre, Zhejiang University, Hangzhou, China

**Keywords:** maize, serotonin N-acetyltransferase, melatonin, drought stress, heat stress

## Abstract

**Introduction:**

Melatonin, a tryptophan-derived indoleamine metabolite with important roles in plant growth and defense, has recently been regarded as a new plant hormone. Maize is one of the most important cereal crops in the world. Although the melatonin receptor gene, *ZmPMTR1*, has already been identified, the genetic basis of melatonin biosynthesis in maize has still not been elucidated. *Serotonin N-acetyltransferase* (SNAT) is the enzyme that converts serotonin to N-acetylserotonin (NAS) or 5-methoxytryptamine (5MT) to melatonin in Arabidopsis and rice, but no SNAT encoding gene has been identified yet in maize.

**Methods:**

The bioinformatics analysis was used to identify maize SNAT genes and the enzyme activity of the recombinant proteins was determined through in vitro assay. The expression levels of *ZmSNAT1* and *ZmSNAT3* under drought and heat stresses were revealed by public RNA-seq datasets and qRT-PCR analysis.

**Results:**

We first identified three maize SNAT genes, ZmSNAT1, ZmSNAT2, and ZmSNAT3, through bioinformatics analysis, and demonstrated that ZmSNAT2 was present in only eight of the 26 cultivars analyzed. We then determined the enzyme activity of ZmSNAT1 and ZmSNAT3 using their recombinant proteins through in vitro assay. The results showed that both ZmSNAT1 and ZmSNAT3 could convert serotonin to NAS and 5-MT to melatonin. Recombinant ZmSNAT1 catalyzed serotonin into NAS with a higher catalytic activity (*K*_m_, 8.6 mM; *V*_max_, 4050 pmol/min/mg protein) than ZmSNAT3 (*K*_m_, 11.51 mM; *V*_max_, 142 pmol/min/mg protein). We further demonstrated that the 228th amino acid Tyr (Y228) was essential for the enzymatic activity of ZmSNAT1. Finally, we revealed that the expression of ZmSNAT1 and ZmSNAT3 varied among different maize cultivars and different tissues of a plant, and was responsive to drought and heat stresses.

**Discussion:**

In summary, the present study identified and characterized the first two functional SNAT genes in maize, laying the foundation for further research on melatonin biosynthesis and its regulatory role in plant growth and response to abiotic stresses.

## Introduction

Melatonin is a tryptophan-derived indoleamine metabolite that has been extensively studied for its important physiological roles in animals such as circadian rhythm, sleep, mood and immune response (Pieri et al., 1994; Rodriguez et al., 2004; Jan et al., 2009; Tan et al., 2015). Since its discovery in plants, melatonin has been proven to be involved in a wide range of plant developmental processes, stress responses, as well as in post-harvest storage, which has led to the recognition of melatonin as a novel phytohormone (Arnao and Hernández-Ruiz, 2020; Tan et al., 2012). The melatonin biosynthesis pathway is thought to be conserved among plants, consisting of four steps, (i) decarboxylation of tryptophan to form tryptamine by tryptophan decarboxylase (TDC); (ii) synthesis of serotonin (5-hydroxytryptamine) from tryptamine by tryptamine 5-hydroxylase (T5H), (iii) conversion of serotonin to N-acetyl-serotonin (NAS) by serotonin N-acetyltransferase (SNAT); and (iv) NAS converted to melatonin catalyzed by NAS methyltransferase (ASMT) and caffeic acid O-methyltransferase (COMT) (Back, 2021). Serotonin can also be converted first to 5-methoxytryptamine (5-MT) and then to melatonin by SNAT.

SNAT is the rate-limiting enzyme in the melatonin biosynthesis pathway. It belongs to the family of General Control Non-repressible 5 (GCN5)-related N-acetyltransferases (GNAT). Two rice genes encoding SNAT, *OsSNAT1* (Kang et al., 2012) and *OsSNAT2* (Byeon et al., 2016) have been cloned and their catalytic activity was validated using recombinant proteins through *in vitro* assays. The amino acid sequences of OsSNAT1 and OsSNAT2 are quite divergent, with only 39% identity and 60% similarity. Phylogenetic analysis indicates that OsSNAT1 and OsSNAT2 are distantly related, suggesting their independent evolution from cyanobacteria before the endosymbiotic event (Byeon et al., 2016). In addition, two SNAT-encoding genes (*AtSNAT1* and *AtSNAT2*) have also been identified in *Arabidopsis thaliana* (Lee et al., 2014, 2019). Based on sequence alignments with rice and/or Arabidopsis SNATs, one or two SNAT genes each have been identified in the following plant species: *Hypericum perforatum* (Zhou et al., 2021), *Pinus taeda* (Park et al., 2014), apple (Wang et al., 2017), grape (Yu et al., 2019), raspberry (Zheng et al., 2021), *Nicotiana benthamiana* (Lee et al., 2021), soybean (Kumar et al., 2022), and cotton (Zhang et al., 2022). Recently, Wang et al. (2022) identified a total of nine homologous genes (named *AtSNAT1*-*AtSNAT9*) in Arabidopsis by searching the apple (*Malus zumi*) SNAT amino acid sequences (Wang et al., 2017), with *AtSNAT8* being *SNAT1* from Lee et al. (2014), and *AtSNAT9* being *SNAT2* from Lee et al. (2019). They found that *AtSNAT6* was highly expressed in the dark and its recombinant protein had a high SNAT activity *in vitro* assay.

Maize (*Zea mays* L.) is one of the most important crops worldwide. The melatonin receptor gene in maize, *ZmPMTR1*, has recently been identified (Khan et al., 2023), and the application of exogenous melatonin has been proven to be beneficial for ameliorating drought stress in maize via alleviation of growth inhibition (Guo et al., 2021), increase of photosynthetic activity and reduction of oxidative damage (Huang et al., 2019). However, not a single gene in the melatonin biosynthesis pathway has been identified in maize. In this study, we first identified SNAT homologous genes and investigated their presence across different cultivars of maize; we then investigated the enzymatic activity of two maize SNATs and the amino acid critical for substrate recognition by using their recombinant proteins; and finally, we investigated their transcriptional response to drought and heat stresses.

## Results

### Identification of maize SNAT candidate genes

**Figure 1:**
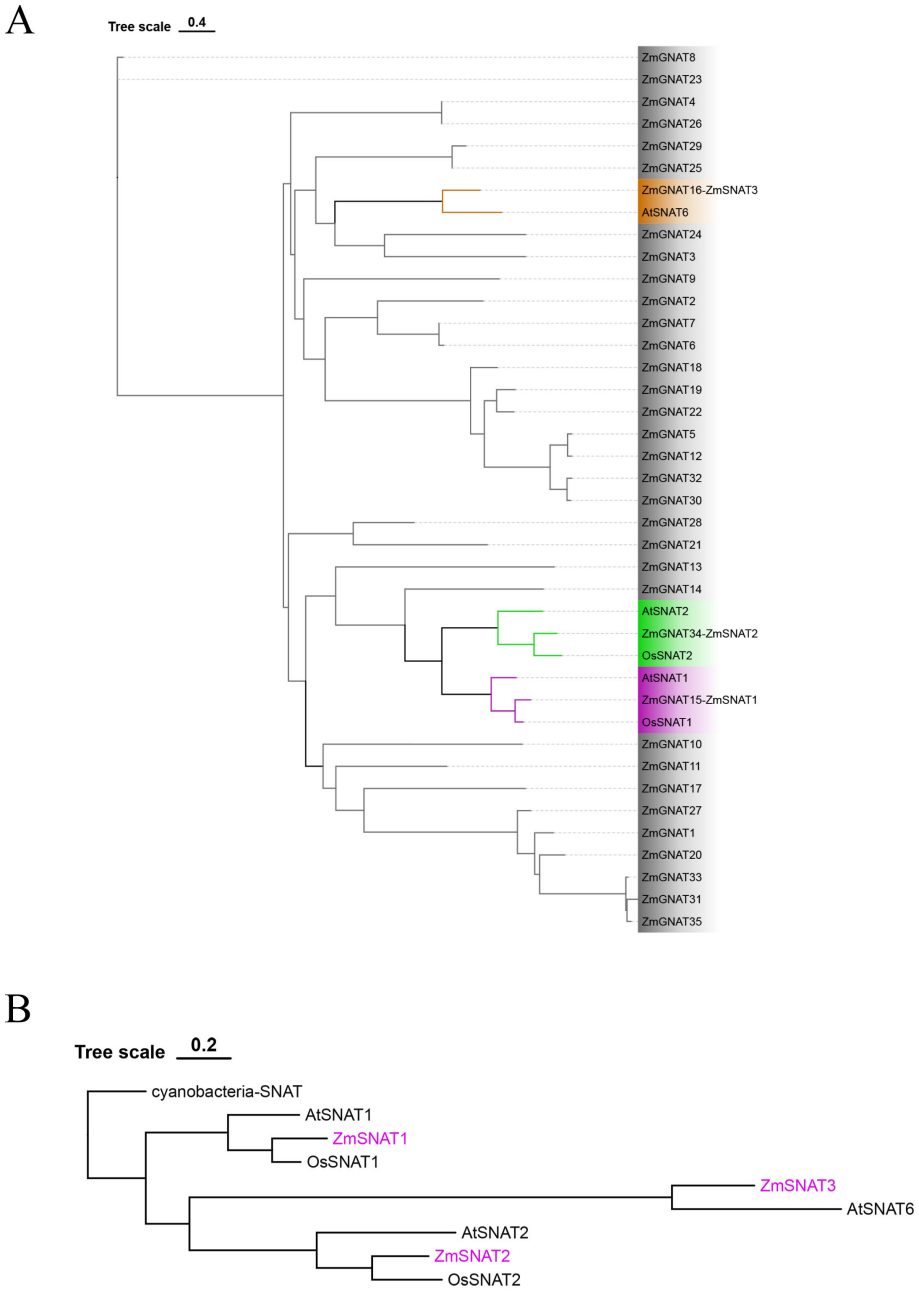
Phylogenetic tree of ZmGNATs and other cloned SNATs. **(A)** ZmGNATs of the *Ki3* genome and SNATs in Arabidopsis and rice; **(B)** Phylogenetic tree of ZmSNATs and SNATs in other species.

Because SNAT belongs to the GNAT family, we hence first searched the GNAT members in the genome of maize cultivar *Ki3*. We then constructed a phylogenetic tree for the ZmGNAT genes together with OsSNAT1, OsSNAT2, AtSNAT1, AtSNAT2 and AtSNAT6 (**Figure 1A**).

A total of 37 GNATs were identified in the *Ki3* genome. Among them, ZmGNAT15, ZmGNAT34, and ZmGNAT16 are closely aligned to OsSNAT1/AtSNAT1, OsSNAT2/AtSNAT2, and AtSNAT6, respectively (**Figure 1A**). Hence, we designated them as ZmSNAT1, ZmSNAT2, and ZmSNAT3 hereafter.

To determine the evolution of maize SNAT genes, we constructed a phylogenetic tree with a cyanobacteria SNAT as the outgroup (**Figure 1B**). Based on this phylogenetic tree, we reason that *ZmSNAT1* probably evolved earlier than *ZmSNAT2*, and *ZmSNAT3* was the latest evolved one (**Figure 1B**).

### Structural characteristics of maize SNAT genes

**Figure 2:**
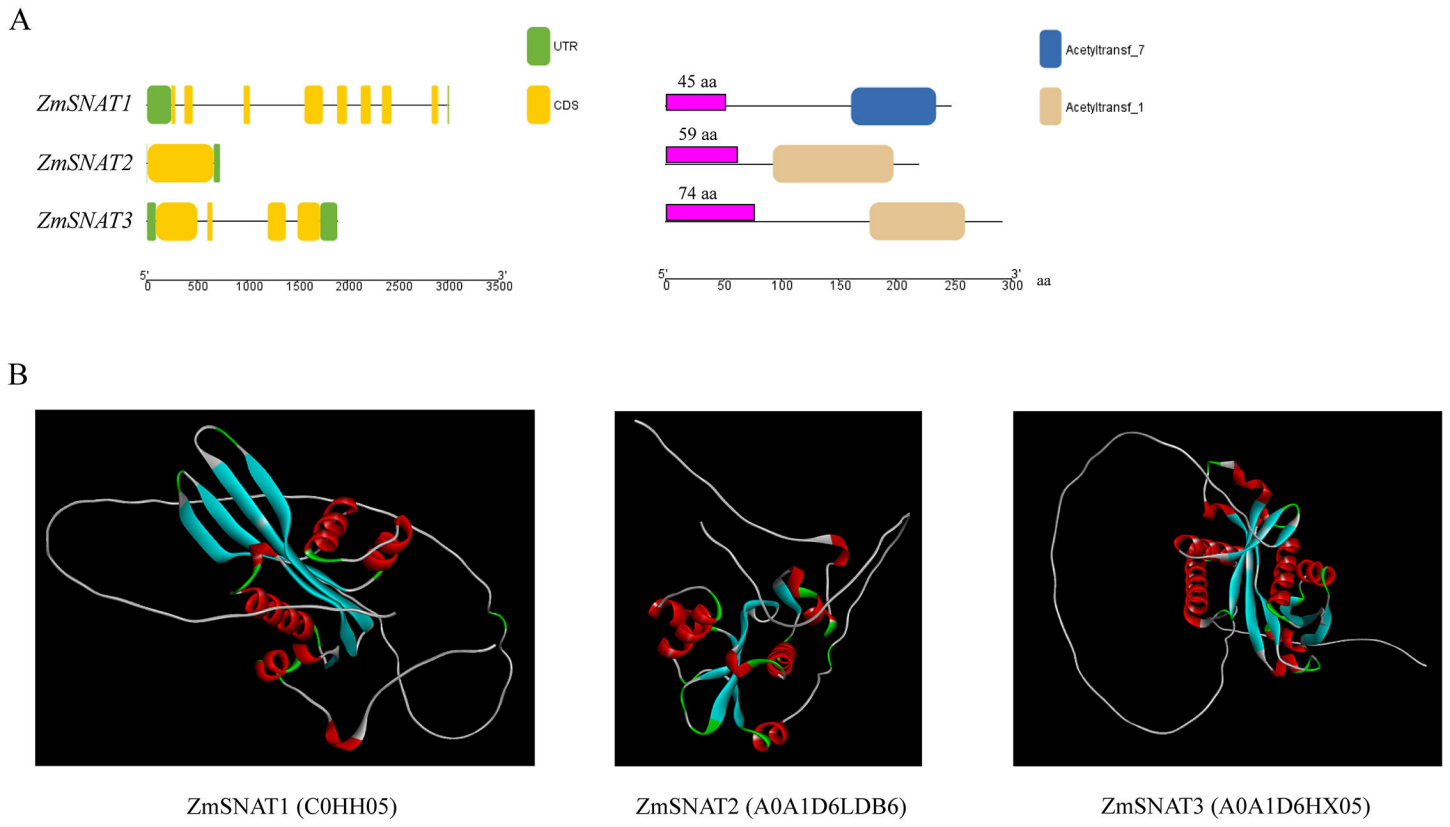
Gene and protein structures of ZmSNATs. **(A)** Gene structures (*left*) and domains (*right*) of ZmSNATs. Untranslated region (UTR) and exon are indicated by the green rectangle and yellow rectangle, respectively. The purple rectangle indicates the predicted chloroplast transit peptide; **(B)** Tertiary structures of ZmSNAT proteins. The structures were constructed on AlphaFold2 with ID in parentheses.

The three maize SNAT genes have very different structures in terms of the number and length of exons, as well as the length of encoded proteins (**Figure 2A**). *ZmSNAT1* harbors 8 exons while *ZmSNAT2* contains only one. The proteins encoded by all three genes contain an acetyltransferase domain of different lengths (**Figure 2A**, *right*). Prediction of protein tertiary structure using AlphaFold2 (AlphaFold Protein Structure Database (ebi.ac.uk) showed the three maize SNATs had strikingly different structures, especially the orientation and helix-sheet-helix architecture (**Figure 2B**).

### Pan-genome-wide presence of ZmSNATs

**Figure 3:**
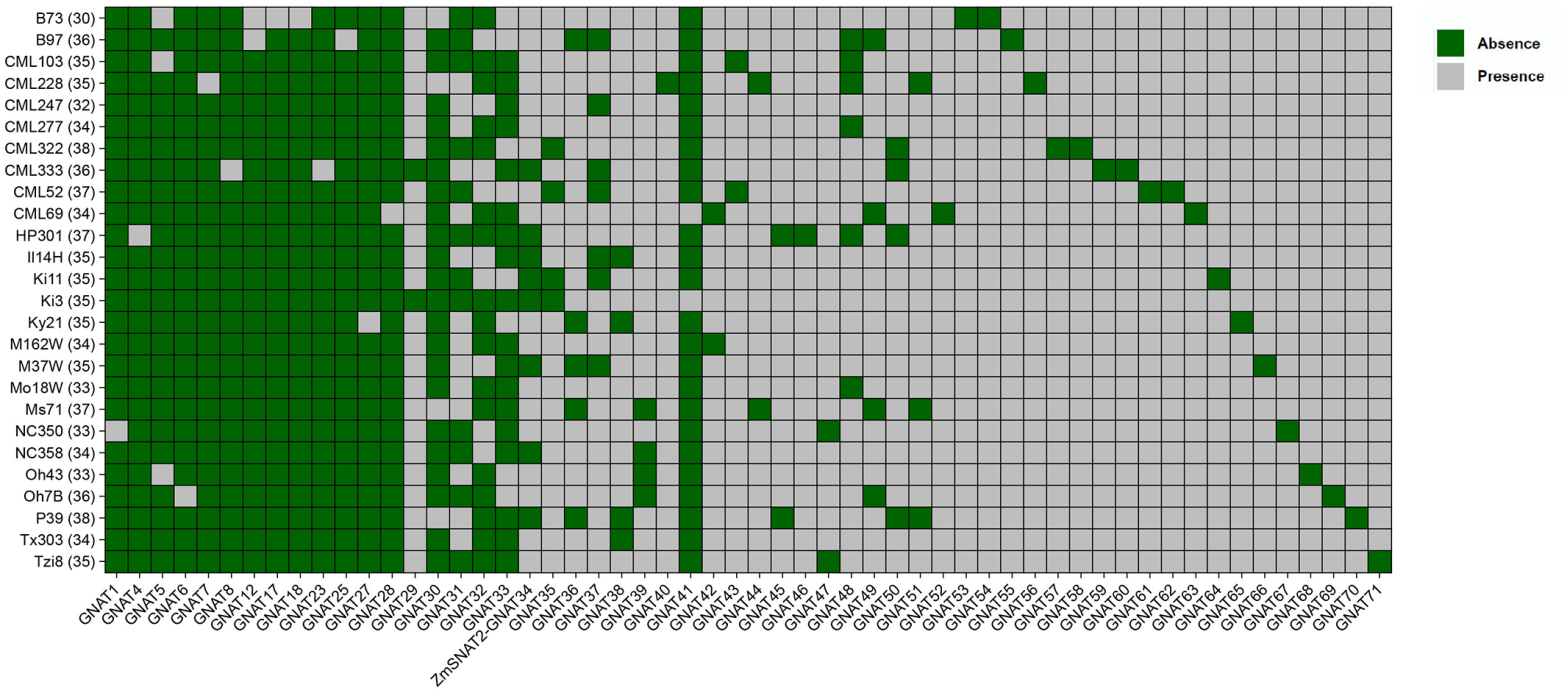
ZmGNAT genes present in 26 maize cultivars. ZmGNAT2, 3, 9-11, 13-16, 19-22,24, 26 are present in all cultivars, hence are not listed. The number in parenthesis of each cultivar is the number of GNAT genes identified in that cultivar.

With the sequencing of more and more maize genomes, the maize pan-genome has been composed of genomes up to 26 maize cultivars (Hufford et al., 2021). To figure out the presence of ZmSNAT genes in different maize cultivars, we first performed a search of the ZmGNATs in each of the 26 cultivars (**Figure 3**; **Supplementary File 1**). The results show that there was a total of 71 ZmGNAT homologous genes across the 26 cultivars, each with 30 (*B73*) to 38 (*CML322*, *P39*) genes (**Figure 3**). Thirty GNAT genes are highly conserved across the 26 cultivars, they are present at least in 24 cultivars; Twenty GNAT genes (ZmGNAT52-71) are present only in one cultivar, while others are present in two or more cultivars. While *ZmSNAT1* and *ZmSNAT3* are present in all the cultivars, *ZmSNAT2* (*ZmGNAT34*) is present in only eight cultivars (**Figure 3**).

### Enzymatic activity of ZmSNAT1 and ZmSNAT3

Because ZmSNAT2 is present in merely eight cultivars, enzymatic activity assay was performed only for ZmSNAT1 and ZmSNAT3.

**Figure 4:**
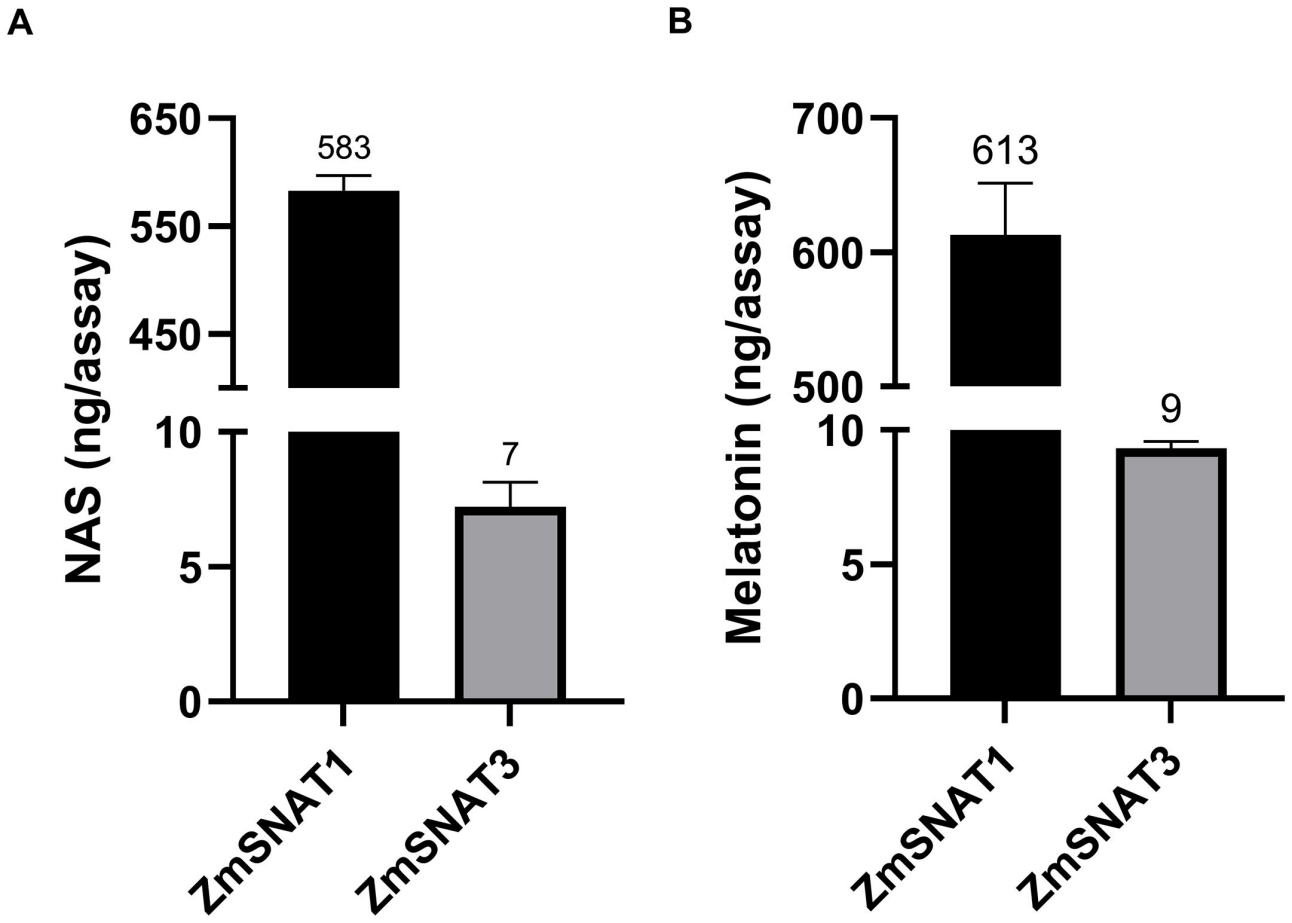
Enzymatic activity of the recombinant ZmSNAT1 and ZmSNAT3, with the substrate of serotonin **(A)** and 5-MT **(B)**. All assays were performed in a total volume of 100 µL PBS (pH = 8.0) with 50 µg the recombinant ZmSNAT1 or ZmSNAT3, and 0.5 mM serotonin **(A)** or 5-MT **(B)**, the reaction was performed at 45°C for 3 hours. Data are shown as mean ± SD, n=3.

Recombinant ZmSNAT1 and ZmSNAT3 were tested *in vitro* using either serotonin or 5-MT as substrate. As shown in **Figure 4**, both proteins catalyzed the production of NAS from serotonin or melatonin from 5-MT. The activity of ZmSNAT1 was 83 (serotonin to NAS) and 68 (5-MT to melatonin) fold higher than that of ZmSNAT3.

**Figure 5:**
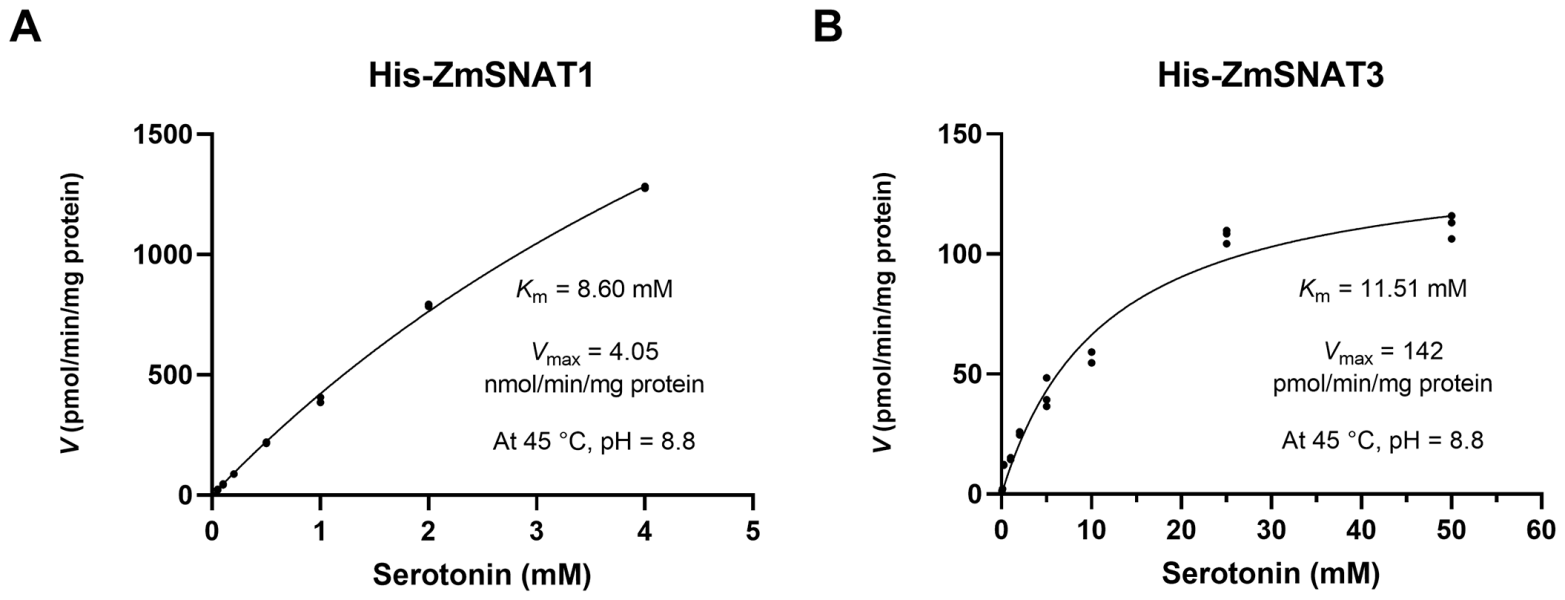
Kinetic parameters of the recombinant ZmSNAT1 and ZmSNAT3. The reaction was performed in PBS buffer (pH = 8.8) with 0.5 mmol/L acetyl-CoA and a series of the concentrations of serotonin for 30 minutes at 45°C. The *K*_m_ and the *V*_max_ were determined using Michaelis-Menten kinetics.

The recombinant ZmSNAT1 with the MBP tag had a low enzymatic activity, with a *K*_m_ value of 4.90 mM and a *V*_max_ value of 173.8 pmol/min/mg, for catalyzing the conversion of serotonin to NAS (**Supplementary Figure S1**). To exclude the MBP tag effect, ZmSNAT1 and ZmSNAT3 were fused with a smaller His-tag for testing for their enzyme kinetics. The results showed that His-ZmSNAT1 had a *K*_m_ of 8.6 mM and a *V*_max_ of 4.05 nmol/min/mg protein (**Figure 5A**). Correspondingly, His-ZmSNAT3 had a *K*_m_ of 11.51 mM) and a *V*_max_ of 142 pmol/min/mg protein (**Figure 5B**), in consistent with the above observation that ZmSNAT3 had a weaker catalytic activity of converting serotonin to NAS than ZmSNAT1.

### The amino acid essential for the enzymatic activity of ZmSNAT1

**Figure 6:**
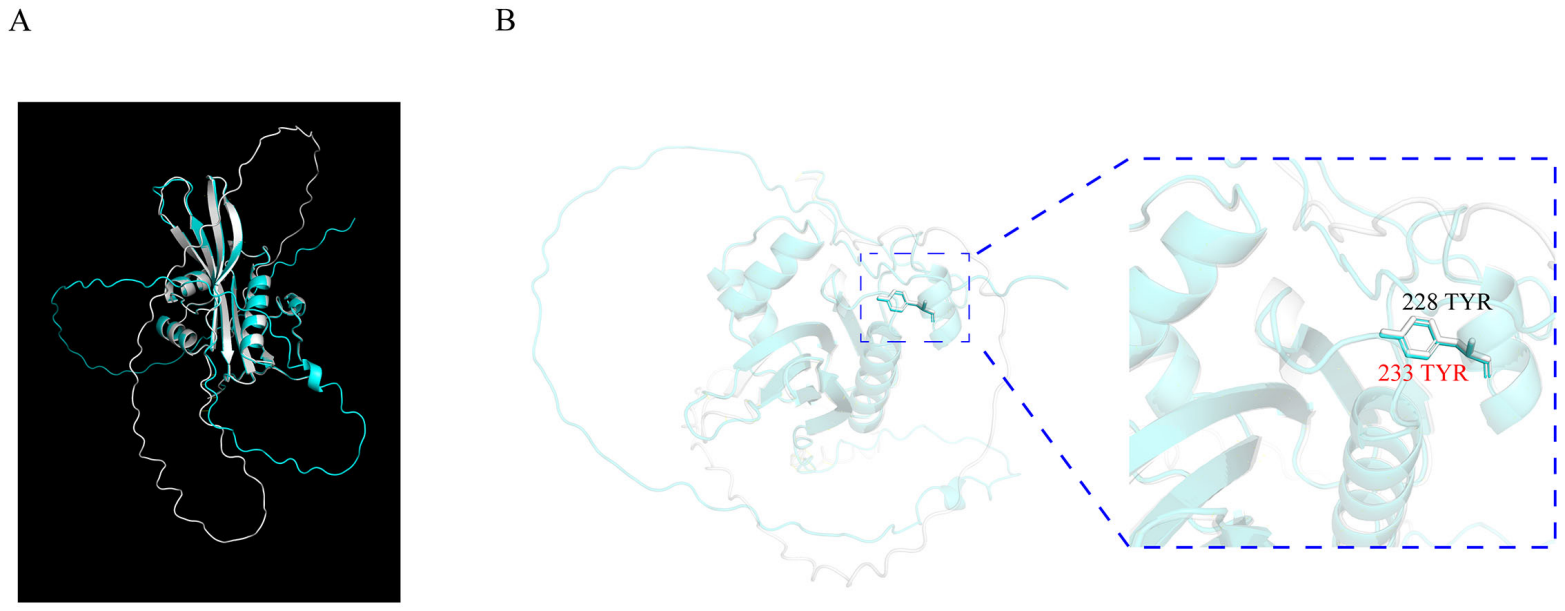
Tertiary structure alignment of OsSNAT1 and ZmSNAT1. **(A)** The overall alignment of OsSNAT1 (cyan) and ZmSNAT1 (white) proteins; **(B)** The enlarged view of 233 TYR (in red) of OsSNAT1 overlapping 228 TYR (in black) of ZmSNAT1. The structure of OsSNAT1 was downloaded from PDBe with ID code 7DAI. The alignment was performed in Pymol.

It was reported that the amino acid Y233 of OsSNAT1 is essential for its function during acetyl-transfer reaction because the replacement of Y233 with Ala or Phe could result in the abolishment of enzymatic activity (Liao et al., 2021). To identify the amino acids in ZmSNAT1 essential for its enzymatic activity, we first performed a tertiary structure comparison between OsSNAT1 and ZmSNAT1, the results showed the tertiary structures of the two proteins are almost identical with an RMSD of 0.312 (**Figure 6A**). Further analysis revealed that Y228 of ZmSNAT1 corresponds to Y233 of OsSNAT1 in the *CML228* cultivar (**Figure 6B**).

**Figure 7:**
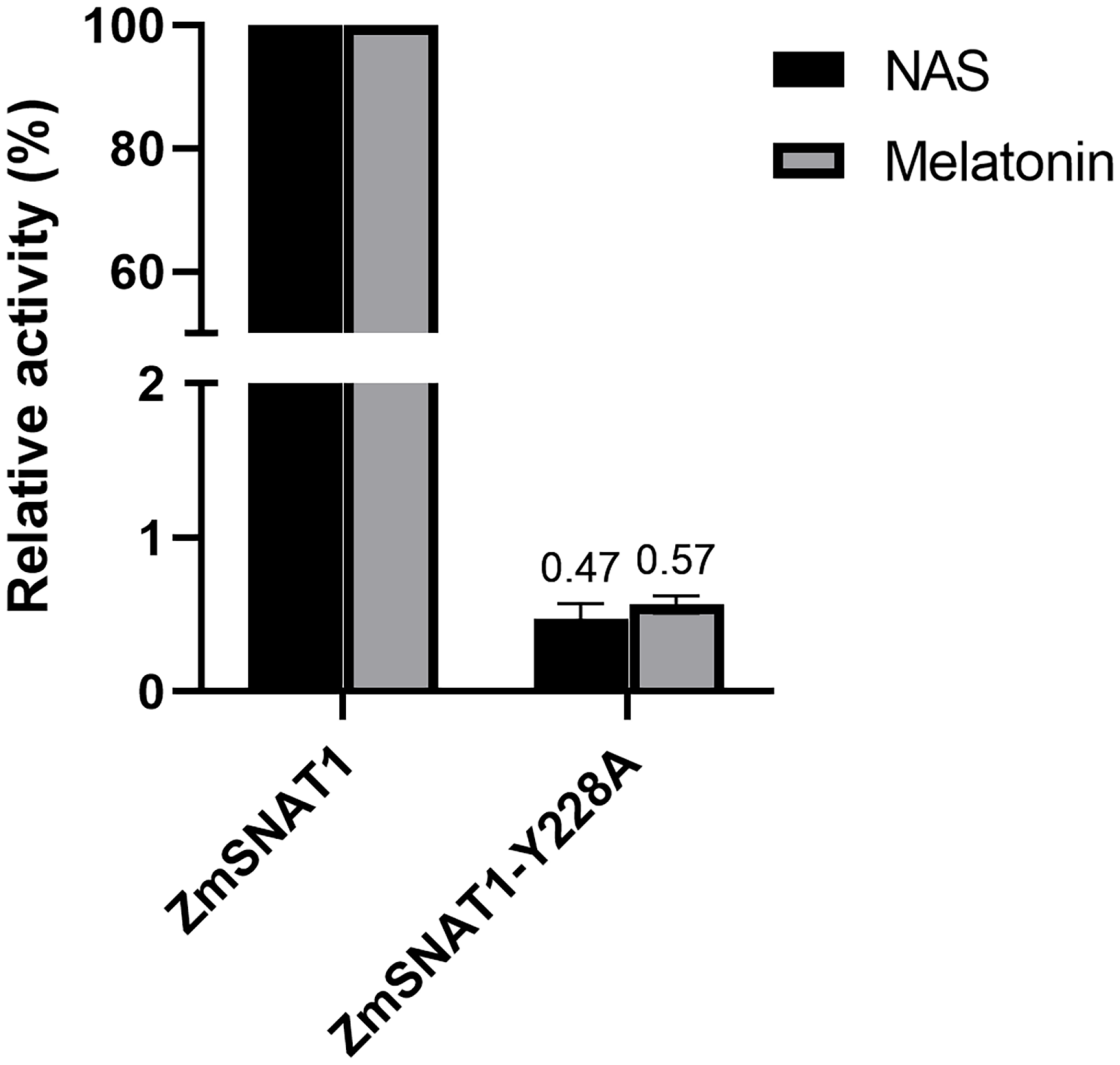
Relative catalytic activities of wild-type ZmSNAT1 and its variant Y228A converting serotonin (dark) to NAS and 5-MT (gray) to melatonin. The activities of the ZmSNAT1 are set to 100%. Each bar represents the mean value of triplicate experiments ± SD (n=3).

To test whether Y228 is truly important, site-directed mutagenesis was applied to generate a mutated ZmSNAT1-Y228A protein. *In vitro* analysis showed that the Y228A mutation almost abolished its enzymatic activity both for converting serotonin to NAS and for 5-MT to melatonin (**Figure 7**). These results indicate that the Y228 is essential for ZmSNAT1.

### Transcription of *ZmSNAT1* and *ZmSNAT3* varied among maize cultivars and in various tissues

**Figure 8:**
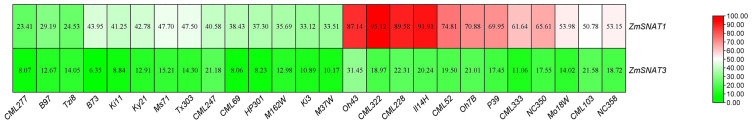
The expression levels of *ZmSNAT1* and *ZmSNAT3* in 26 maize cultivars. Data were extracted from ENA AaaryExpress E-MTAB-8633 and E-MTAB-8628 generated by Hufford et al. (2021).

To have a general view of the transcription of *ZmSNAT1* and *ZmSNAT3* in maize, we first investigated the RNA-seq data of 26 maize cultivars generated by Hufford et al. (2021). Overall, *ZmSNAT1* seemed to have a greater transcription level than *ZmSNAT3*. For instance, the FPKM of *ZmSNAT1* was ~1.7-6.9 fold greater than that of *ZmSNAT3* in the eleventh leaves (**Figure 8**). Furthermore, there were also variations of transcription of both *ZmSNAT1* and *ZmSNAT3* among the 26 cultivars tested, with the FPKM value varied from 23.41 to (*CML277*) to 95.12 (*CML232*) for *ZmSNAT1*, and from 6.35 (*B73*) to 31.45 (*Oh43*) for *ZmSNAT3* (**Figure 8**).

**Figure 9:**
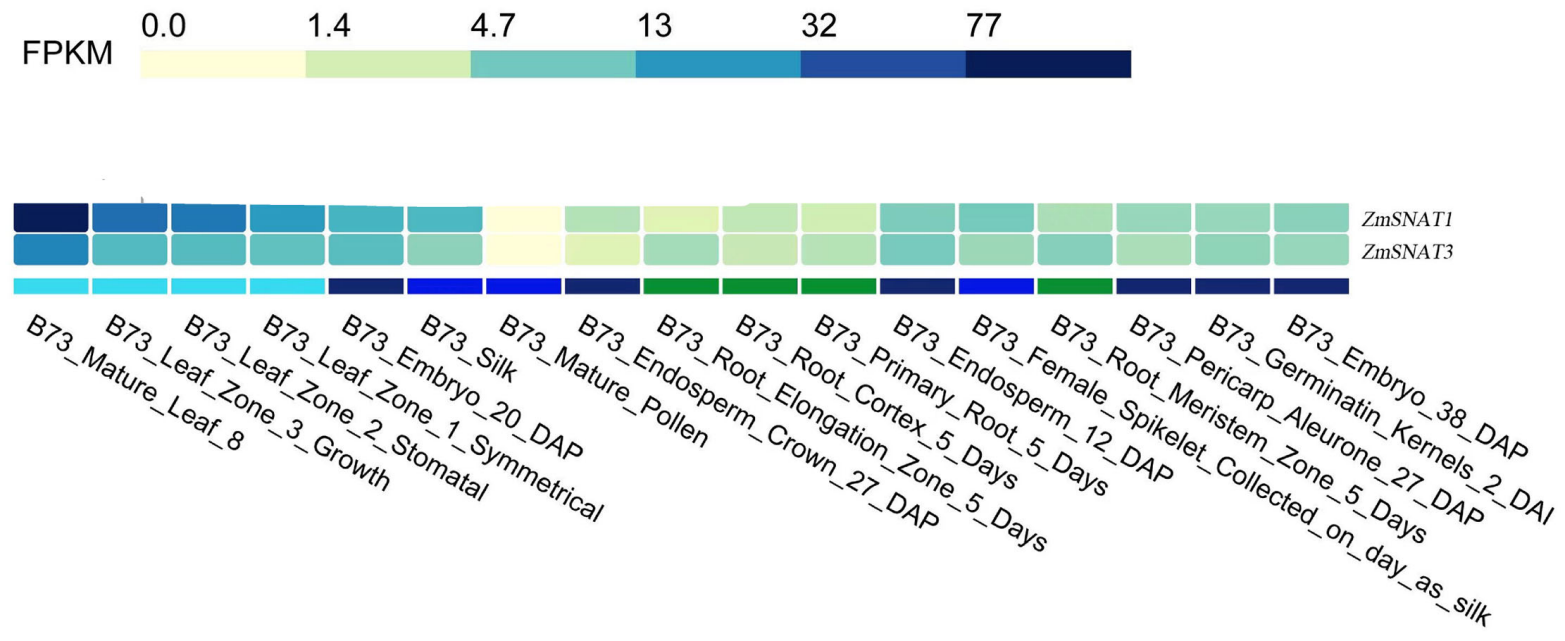
Transcription of *ZmSNAT1* and *ZmSNAT3* in different tissues of a maize plant (cultivar *B73*, unit: FPKM). Data were extracted from the ZEAMAP database.

We also determined the expression pattern of *ZmSNAT1* and *ZmSNAT3* across various tissues using the RNA-seq data of the cultivar *B73* collected in the ZEAMAP database. Overall, both genes were highly expressed in leaves, particularly in mature leaves, and to a lesser extent in other tissues (embryos, silk, developing and mature seeds, etc.). They are barely expressed in mature pollen and roots (**Figure 9**). Comparatively, *ZmSNAT1* had greater expression than *ZmSNAT3* in most tissues except roots.

### Transcription of *ZmSNAT1* and *ZmSNAT3* was responsive to drought and heat stress

**Figure 10:**
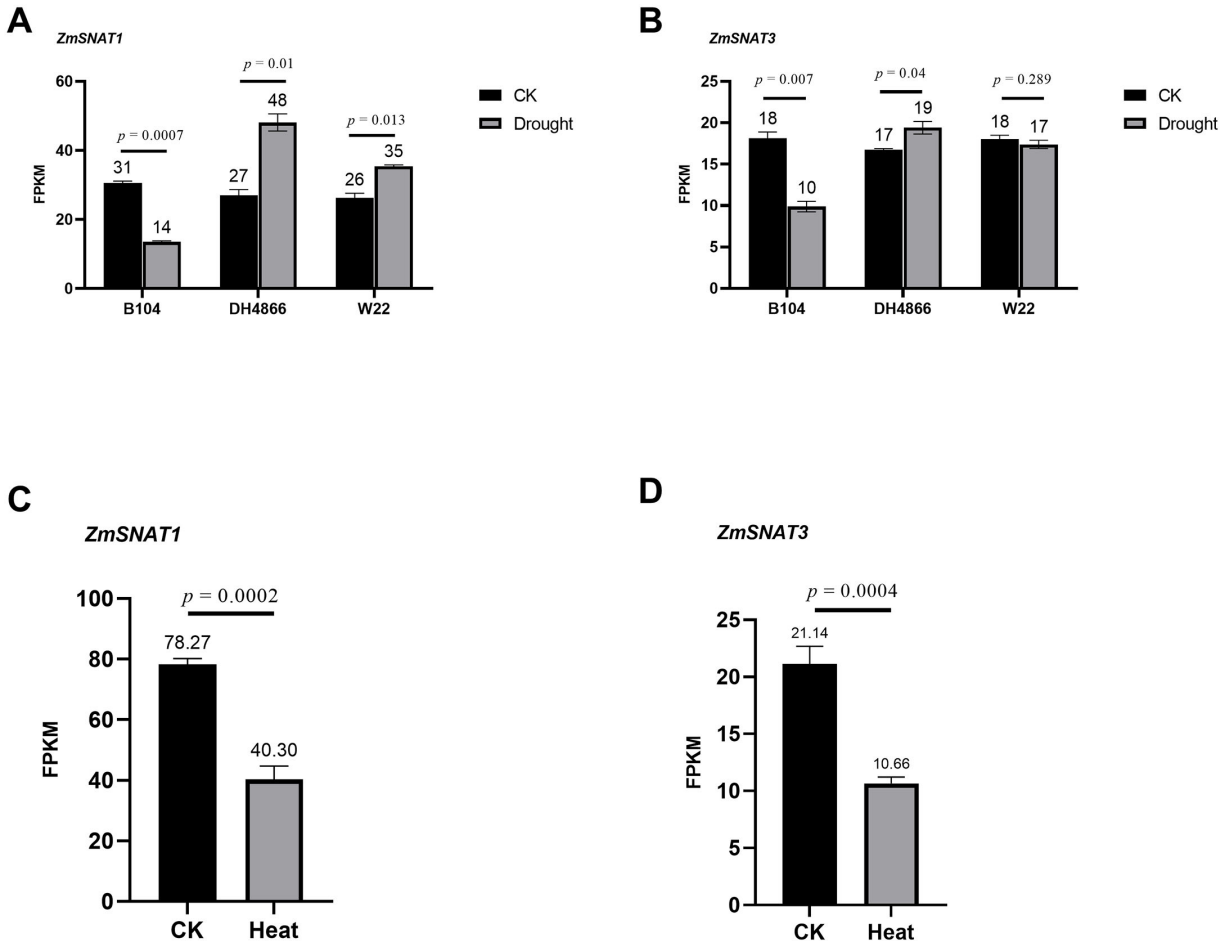
Transcription of *ZmSNAT1* and *ZmSNAT3* in maize plants subjected to drought and heat stress. The FPKM values of *ZmSNAT1* **(A)** and *ZmSNAT3* **(B)** in response to drought stress in the cultivars *B104*, *DH4866* and *W22*; **(C, D)** The FPKM values of *ZmSNAT1* and *ZmSNAT3* in response to hear stress in the cultivar *W22*. The datasets analyzed for drought and heat stress were downloaded from the GEO database with the accession GSE137780 and GSE268429, respectively. Each bar represents the mean value of triplicate experiments ± SD (n=3).

It is known that melatonin content is increased upon certain stresses in maize (Colombage et al., 2023). Therefore, we investigated the expression levels of *ZmSNAT1* and *ZmSNAT3* in the leaves of plants subjected to drought and heat stresses. First, we analyzed the RNA-seq data of three cultivars available in the GEO database. The results showed that *ZmSNAT1* was significantly upregulated in the cultivars *DH4866* and *W22*, but downregulated in *B104* when subjected to a 5-day drought stress (**Figure 10A**). *ZmSNAT3* was also significantly upregulated in *DH4866* and downregulated in *B104*, but no significant changes were observed in *W22* (**Figure 10B**). After being subjected to heat stress (39°C, 1 hour), both *ZmSNAT1* and *ZmSNAT3* were significantly down-regulated in leaves of the *W22* cultivar (**Figures 10C, D**).

**Figure 11:**
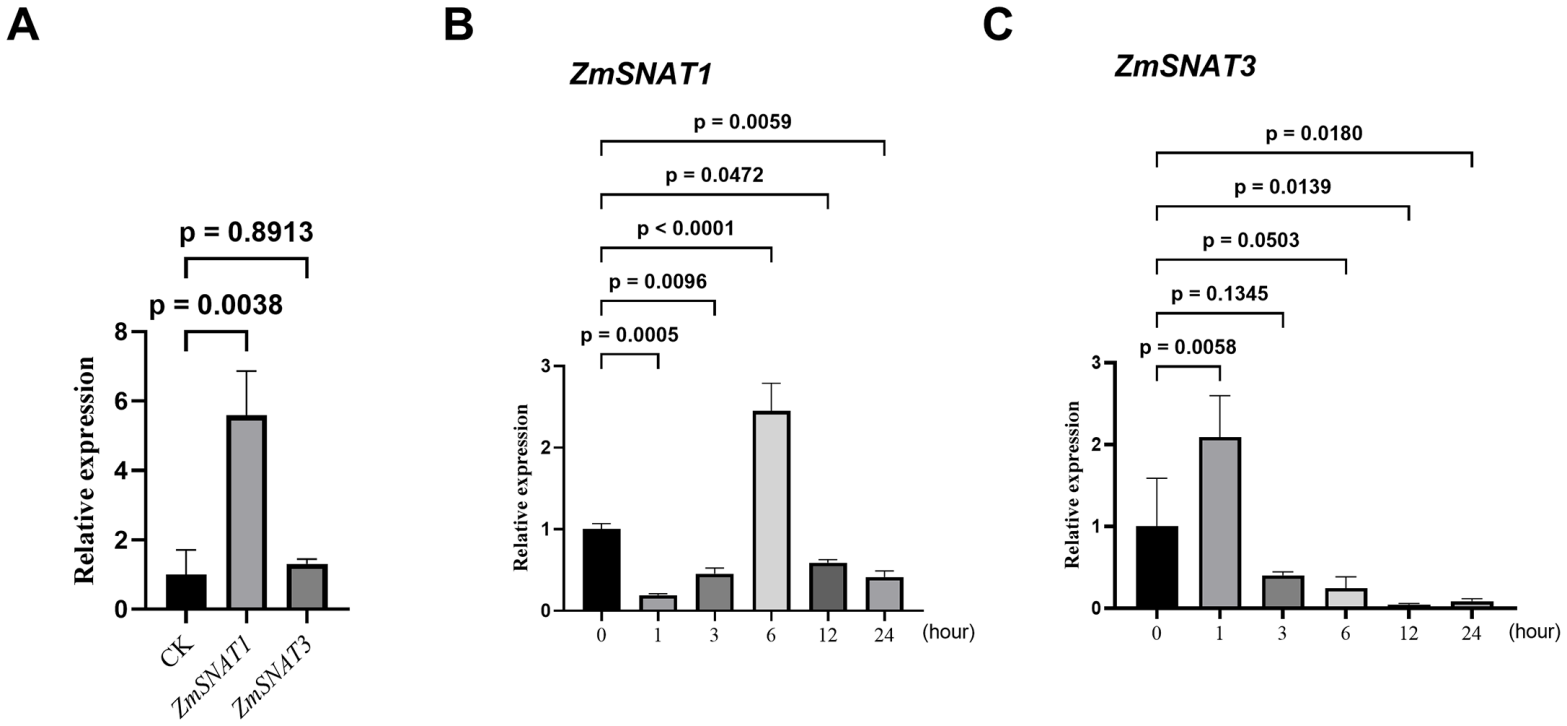
The relative expression levels of *ZmSNAT1* and *ZmSNAT3* in plants of maize cultivar *W22* subjected to drought stress **(A)** and heat stress **(B, C)**. Each bar represents the mean value of triplicate experiments ± SD (n=3). The maize TUB-ribosylation factor gene was used as an internal control. The relative expression levels were calculated using the comparative 2^-ΔΔCt^ method (Livak and Schmittgen, 2001).

We further performed indoor experiments to investigate the transcriptional responses to drought and heat stresses, using three-leaf seedlings of *W22*. QRT-PCR analysis showed that, after being subjected to drought stress, *ZmSNAT1* was significantly up-regulated, with a 5.5-folder increase of transcript abundance, while *ZmSNAT3* was not (**Figure 11A**). After being subject to heat stress (45°C), *ZmSNAT1* was first significantly down-regulated (1, 3 h), then significantly upregulated (6 h), and again downregulated (12, 24 h) (**Figure 11B**). *ZmSNAT3* was significantly up-regulated soon after heat stress (1 h, +110%), and significantly downregulated afterwards, with mRNA abundance being reduced to 40.2%, 24.6%, 4. 7% and 8.7% that of the control at 3, 6, 12 and 24 h, respectively (**Figure 11C**).

## Discussion

Previous studies have already shown that the application of exogenous melatonin could promote plant growth and development, and ameliorate damages caused by abiotic stresses. However, little is known about melatonin biosynthesis, and its regulation and response to abiotic stresses in maize plants. The present study first identified three maize SNAT genes that are homologous to cloned SNAT genes of either rice or Arabidopsis or both. Two of them, *ZmSNAT1* and *ZmSNAT3*, are present in all 26 maize cultivars, while *ZmSNAT2* is present only in eight cultivars. This study further demonstrated that while both ZmSNAT1 and ZmSNAT3 could catalyze melatonin biosynthesis, ZmSNAT1 had an enzymatic activity far greater than ZmSNAT3. Furthermore, this study determined an amino acid in ZmSNAT1 essential for its enzymatic activity. At last, this study demonstrated that transcription of *ZmSNAT1* and *ZmSNAT3* varied among different maize cultivars and different tissues, and was responsive to drought and heat stresses. The above findings hence laid a solid foundation for further research on the genetics, biology and evolution of melatonin in maize.

### More SNAT genes in maize

Through the homolog search of rice and Arabidopsis SNAT genes, we identified 3 SNAT homologs in maize in this study. However, we believe that there might be more SNAT genes in maize to be identified. The plant SNAT gene was first identified in rice (Kang et al., 2012), which was later used directly for the search for homologs in other plant species including Arabidopsis (Lee et al., 2014, 2019). The Arabidopsis SNATs were also later used for search of homologs in dicots (Wang et al., 2017). Because the two rice SNATs had a low identity and similarity, suggesting that there might be more SNATs to be identified in rice and other plant species. Indeed, Wang et al. (2022) identified 9 SNAT genes in Arabidopsis (including two that had already been functionally validated) through a homolog search of an apple SNAT, though they only validated the anticipated enzymatic function for one of them.

The results of the present study also suggest there might be more SNAT genes in maize based on the following evidence. Firstly, only ZmSNAT1 and ZmSNAT2 were aligned to rice SNATs, while ZmSNAT3 was aligned to a newly identified Arabidopsis SNAT6 (**Figure 1**), suggesting that it is likely to identify the new SNATs in maize when new SNAT genes identified in other plants are used for search. Secondly, the presence of ZmSNAT2 in only 8 of the 26 maize cultivars, together with the presence of various GNATs only in a single cultivar (**Figure 3**), also suggests that more cultivar-specific SNATs might exist. Thirdly, it is known that melatonin is in mitochondria in animals (Reiter et al., 2022). However, this study only identified SNATs with a chloroplast transit peptide (**Figure 2B**), and hence presumably to be located in chloroplast (see Materials and Methods). Because mitochondrion is known to be an important organelle, particularly in stress response, where melatonin plays an important role, it is quite plausible that there are new genes encoding mitochondrial SNATs in maize yet to be identified.

### Enzymatic characteristics of ZmSNATs

The present study demonstrated that both ZmSNAT1 and ZmSNAT3 could use serotonin or 5-MT as the substrate to produce NAS or melatonin, which is consistent with the characteristics of other plant SNATs (Lee et al., 2014). It was revealed that ZmSNAT1 had an enzymatic activity far higher than ZmSNAT3, although the underlying mechanism remains to be investigated.

The kinetic parameters of the recombinant proteins showed that ZmSNAT1 and ZmSNAT3 had the enzymatic activity similar to those of rice and Arabidopsis. For instance, ZmSNAT1 had a *K*_m_ value (8.60 mM), which is ~ 20 folder greater than that of AtSNAT1 (309 µM) (Lee et al., 2014) and OsSNAT1 (385 µM) (Kang et al., 2012), but its *V*_max_ value (4.05 nmol/min/mg) (**Figure 5A**) is also greater than that of AtSNAT1 (1.4 nmol/min/mg) (Lee et al., 2014) and OsSNAT1 (282 pmol/min/mg) (Kang et al., 2012). Based on the *K*_m_ value ZmSNAT3 (*K*_m_ 11.51 mM) seemed to have weaker substrate binding ability than AtSNAT6 (*K*_m_ = 5 mM), however, its *V*_max_ value (142 pmol/min/mg) is very similar to that of AtSNAT6 (147 pmol/min/mg) (**Figure 5B**), indicating ZmSNAT3 also had a catalytic rate similar to AtSNAT6. The reasons why the maize SNATs had higher *K*_m_ values than rice and Arabidopsis homologs but still had greater or similar *V*_max_ values remain to be investigated.

We further determined Y228 in ZmSNAT1 to be the amino acid essential for its enzymatic activity. This was achieved by the alignment of the protein tertiary structures between ZmSNAT1 and OsSNAT1, of which the crystal structure and the essential amino acids were already reported (Liao et al., 2021). Further analysis revealed that Y228 is well conserved in all 26 maize cultivars analyzed (**Supplementary Figure S2**), which also corroborates the importance of this key residue.

### Responsive transcription of *ZmSNATs* to abiotic stresses

Due to climate change, plants are experiencing more and ever-increasing stresses such as heat and drought (Li et al., 2023; Wang et al., 2023). It has already been well proven that melatonin could alleviate growth inhibition and damage caused by drought and other abiotic stresses (Huang et al., 2019). Application of exogenous melatonin can reduce ROS accumulation and improve the efficiency of photosystem and stomatal conductance to enhance drought tolerance (Ye et al., 2016; Fleta-Soriano et al., 2017; Huang et al., 2019; Su et al., 2019) and thermotolerance (Ahammed et al., 2019). By analyzing the public available maize transcriptome datasets, we revealed that the transcription of *ZmSNAT1* and *ZmSNAT3* was responsive to drought and heat stresses (**Figure 11**). When subject to drought stress, the transcription of *ZmSNAT1* and *ZmSNAT3* was significantly downregulated in one cultivar but upregulated in the other two cultivars. A similar observation was reported in *Hypericum perforatum*, where the expression level of *HpSNAT1* reached the highest at 8 h after drought stress (Zhou et al., 2021). In one cultivar tested for heat stress, both genes were significantly downregulated (**Figure 10**).

We further determined the transcription of *ZmSNAT1* and *ZmSNAT3* in the *W22* cultivar subjected to heat or drought stresses by qRT-PCR analysis. Drought stress activated the transcription of *ZmSNAT1* but not *ZmSNAT3* (**Figure 11A**), which is consistent with the results of transcriptome analysis. On the other hand, we observed time-dependent effect of heat stress on the transcription of *ZmSNAT1* and *ZmSNAT3* (**Figures 11B, C**). All these observations suggest the transcription of *ZmSNAT1* and *ZmSNAT3* is responsive to heat and drought stresses, suggesting melatonin biosynthesis is finely regulated through the transcription of *ZmSNAT1* and *ZmSNAT3*. However, the mechanism(s) underlying this phenomenon and the key players remain to be investigated.

In a few plants, it is already known that the crosstalk between melatonin and other hormones such as SA and ABA is involved in the melatonin-mediated tolerance to drought stress (Li et al., 2015; Wei et al., 2015; Prakash et al., 2019; Arnao and Hernández-Ruiz, 2019; Moustafa-Farag et al., 2020; Tiwari et al., 2021). In tomato seedlings, it was observed that melatonin enhances drought tolerance by stimulating sugar metabolism and negatively regulating ABA synthesis (Jahan et al., 2024). Melatonin biosynthesis under stress could also be regulated through protein interactions. In cotton, for instance, GhSNAT3D might interact with GhASMT and GhSNAT25D to regulate the biosynthesis of melatonin when subjected to different stresses (Zhang et al., 2022). In tomato, a chaperone HSP40 in chloroplasts could stabilize SlSNAT against heat shock-induced degradation (Wang et al., 2020). These findings of melatonin biosynthesis regulation provide useful clues to uncover the mechanisms of melatonin biosynthesis regulation in response to heat and drought stress in maize.

Maize is a worldwide crop and has spread across the world. We observed that there is a great variation of the *ZmSNAT1* and *ZmSNAT3* transcription level among the 26 cultivars (**Figure 8**). It is worthwhile to further investigate whether it is a result of their local adaptation. It is also very intriguing to identify the genetic basis underlying the transcriptional variation for enhancing the resilience of maize cultivars to environmental stresses. Since melatonin has recently been considered as a new plant hormone, it will be worthwhile to dissect the pathways of its biosynthesis, regulation and signaling in maize. The identification and characterization of ZmSNAT1 and ZmSNAT3, though very preliminary, lay the foundation for further studies on identifying new SNATs in maize and other plants as Wang et al. (2022) did in Arabidopsis. The functions of melatonin in maize growth, development, environmental stresses and other biological processes could be investigated by producing knockout/edited/silenced/overexpressed plants, such as in rice (Byeon and Back, 2016; Hwang and Back, 2019, 2020).

## Conclusions

In this study, we identified and validated two maize SNAT genes, *ZmSNAT1* and *ZmSNAT3*, that encode proteins with the function of converting serotonin to NAS or 5-MT to melatonin. *ZmSNAT1* appears to have a greater transcription level and to encode a protein having the enzyme activity greater than that encoded by *ZmSNAT3*. The Y228 residue in ZmSNAT1 is essential for its enzymatic activity and is conserved across maize cultivars. Another maize SNAT gene, ZmSNAT2 is only present in 8 of 26 cultivars analyzed. We also determined the transcription of both *ZmSNAT1* and *ZmSNAT3* is responsive to drought and heat stresses.

## Materials and methods

### Identification of maize GNAT family genes

The genomes of 26 maize cultivars sequenced and assembled by Hufford et al. (2021) were downloaded from maizeGDB (Welcome to MaizeGDB). The hidden Markov model of GNAT domain (PF00583) was used as a query to search the proteins in the genomes of maize cultivars (Yu et al., 2019), with a threshold of e < 1e^-5^ and the length of protein < 500 aa (amino acid).

### Phylogenetic analysis and presence/absence assay of ZmGNAT genes

The protein sequences of ZmGNAT identified above and verified OsSNAT1 (Kang et al., 2012), OsSNAT2 of rice (Byeon et al., 2016) and AtSNAT1 (Kang et al., 2012), AtSNAT2, AtSNAT6 of Arabidopsis (Lee et al., 2014, 2019; Wang et al., 2022) were used for phylogenetic analysis. Multiple sequence alignments were performed using MAFFT version 7 [MAFFT alignment and NJ/UPGMA phylogeny (cbrc.jp)] and the phylogenetic tree was constructed by IQ-TREE web server [IQTREE Web Server: Fast and accurate phylogenetic trees under maximum likelihood (univie.ac.at)] with the default parameter. The presence/absence of ZmGNATs across 26 maize cultivars was generated by TBtools v2.112 (Chen et al., 2023). The heatmap was generated by TBtools v2.112 (Chen et al., 2023).

### Production of ZmSNAT recombinant proteins in *Escherichia coli*

Both ZmSNAT1 and ZmSNAT3 contain a short chloroplast transit peptide (cTP) sequence as predicted by ChloroP (Emanuelsson et al., 1999). To exclude this cTP sequence in recombinant proteins, PCR primers were designed to amplify *ZmSNAT1* or *ZmSNAT3* coding sequences without the cTP portion from maize cultivar *CML322* (**Supplementary Table S1**). The PCR amplicons thus amplified from *ZmSNAT1* and *ZmSNAT3* cDNA were then inserted into the pMAL-C6T vector linearized by *Alwn*I or pET-45b vector linearized by *Kpn*I. The recombinant vectors were then transformed into *E. coli* Transetta (DE3) (TransGen Biotech, Beijing, China). To produce recombinant ZmSNATs proteins, the transformed *E.coli* were cultured on the Luria-Bertani (LB) broth agar (10 g/L Tryptone, 5 g/L yeast extract, 10 g/L NaCl and 15 g/L agar) containing 100 mg/L ampicillin and picked up one colony into 5 mL of Luria-Bertani (LB) broth (10 g/L Tryptone, 5 g/L yeast extract, 10 g/L NaCl) containing 100 mg/L ampicillin and 34 mg/L chloramphenicol and incubated at 37°C overnight. 3 mL overnight culture was inoculated into 300 mL LB broth containing 100 mg/L ampicillin and 34 mg/L chloramphenicol until the optical density of the *E.coli* culture at 600 nm (OD_600_) reached 0.6-0.8. after the addition of 100 µM isopropyl-β-D-thiogalactopyranoside (IPTG; Sigma, St.Louis, MO, USA), the culture was grown at 23°C and shaken at 160 rpm for 14 hours. After breaking up the cells of *E.coli* by the high-pressure homogenizer, the following purification steps using Ni-NTA or amylose resin were performed according to the manufacturer’s instructions (Vazyme, Nanjing, China; NEB, Beijing, China). Purified protein was concentrated through an Amicon ultra-4 centrifugal filter (Merck Millipore, Carrigtwohill, Ireland) and dissolved in PBS (pH 8.0) (**Supplementary Figure S3**).

### Measurement of SNAT enzyme activity

Purified recombinant SNATs were used for enzymatic activity assay. The reaction was performed in a total volume of 100 µL containing 0.5 mM of serotonin or 5-MT as substrate, and 0.5 mM of acetyl-CoA in a PBS buffer (pH 8.0), the mixture was then incubated at 45°C for 3 hours (or various other time periods for the determination of *K*_m_ and *V*_max_) and stopped by the addition of 200 µL methanol (MeOH). The reaction buffers were then centrifuged at 10,000 rpm for 2 min and the supernatants were passed through a PTFE membrane (0.22 µm). A 5 µL aliquot was used for the determination of NAS or melatonin on an HPLC with the fluorescence detector system (LC-20, Shimadzu). All chemicals’ separations were performed at a flow rate of 1 mL/min and separated on the Ultimate XB-C18 column (4.6 x 150 mm; Welch, Shanghai, China) with an isocratic elution with 30% MeOH in 0.1% formic acid for 20 min. NAS and melatonin were detected at 280 nm (excitation) and 348 nm (emission). All measurements were reproduced in triplicate. The enzymatic activities were calculated by subtracting the value of nonenzymatic reaction controls, i.e., reactions with only substrate and 0.5 mM of acetyl-CoA but without ZmSNAT recombinant proteins. The data were fit to the Michaelis-Menten equation using Prism 9 (GraphPad) to obtain the kinetic parameters. All assays were run in triplicate. The concentration of recombinant proteins was determined by the Bradford method using a protein assay dye (Bio-Rad, Hercules, CA, USA).

### Comparison and visualization of protein tertiary structures

The crystal structure of OsSNAT1 (PDBe ID: 7DAI) was downloaded from PDBe [Homepage | Protein Data Bank in Europe (ebi.ac.uk)]. The tertiary structures of ZmSNATs were downloaded from AlphaFold2 (AlphaFold Protein Structure Database (ebi.ac.uk)). The tertiary structure alignment of OsSNAT1 and ZmSNAT1 was performed and the RMSD was calculated by Pymol 2.5.2.

### Source of transcriptomes and visualization of transcription profile

For analysis of the expression profile of *ZmSNAT1* and *ZmSNAT3*, the revelation of tissue-specific expression profile was performed in ZEAMAP [ZEAMAP: a comprehensive database adapted to the maize multi-omics era. (cngb.org)], using the RNA-seq data of *B73*. The dataset of drought stress was downloaded from GSE137780 and the dataset of heat stress from GSE268429. The FPKM values were averaged per treatment per cultivar for *ZmSNAT1* and *ZmSNAT3*. The expression datasets of 26 maize cultivars were obtained from Hufford et al. (2021).

### Plant growth conditions and stress treatment

The maize *W22* cultivar was used in this experiment. Seeds were germinated and grown in Hoagland solution at 28°C for 14 days. Then the plants were grown in pots with the nutritional soil (Peilei, Jiangsu, China) with a soil moisture of ~30% in a greenhouse with an average temperature of 28°C. For drought treatment, three-leaf seedlings were grown in two groups, one group with normal watering (control) and another without watering for 10 days. For heat treatment, three-leaf seedlings were incubated in a phytotron under 45°C and leaves were collected at 0, 1, 3, 6,12 and 24 h, respectively. The plants grown under 28°C were used as the control of each time point. The leaf samples were collected in due time of treatment and were immediately frozen in liquid nitrogen and stored at −80°C until use for RNA extraction.

### Quantitative real-time PCR

For quantitative real-time PCR (qRT-PCR), total RNAs were extracted from maize leaves with the RNAprep Pure Plant Kit (Tiangen, Beijing, China). According to supplier instructions, cDNAs were synthesized using PrimeScriptTM RT Reagent Kit with gDNA Eraser (Tiangen, Beijing, China). The Primer Premier 5.0 was used to design the primers for qRT-PCR (**Supplementary Table S1**), and the maize TUB-ribosylation factor gene was used as an internal control. The reaction was performed on Bio-Rad CFX ConnectTM using SYBR-Green to detect gene expression levels. For all qRT-PCR analyses, triplicate biological samples were collected. Data were analyzed using Bio-Rad CFX Manager software.

### Statistical analysis

Student’s *t*-test and one-way ANOVA were used for the statistical evaluations.

## Data Availability

The original contributions presented in the study are included in the article/**Supplementary Material**. Further inquiries can be directed to the corresponding authors.
