# Supplementary material for: Identification and functional analysis of two serotonin N-acetyltransferase genes in maize and their transcriptional response to abiotic stresses
Source: Front Plant Sci. 2024 Oct 1;15:1478200. doi: 10.3389/fpls.2024.1478200 (PMC11481039; doi:10.3389/fpls.2024.1478200)
Supplement: Supplementary file 2 [file DataSheet2.docx]

**Identification and functional analysis of two *serotonin N-acetyltransferase* genes in maize and their transcriptional response to abiotic stresses**

Xiaohao Guo^1^, Le Ran^1^, Xinyu Huang^1^, Yuanyuan Tan^1,2^ and Qingyao Shu^1,2*^

^1^State Key Laboratory of Rice Biology & Breeding, and Zhejiang Provincial Key Laboratory of Crop Germplasm Innovation and Exploitation, The Advanced Seed Institute, Zhejiang University, Hangzhou, China.

^2^Zhejiang University – Wuxi Xishan Joint Modern Agricultural Research Centre, Zhejiang University, Hangzhou, China.

**Figure S1**


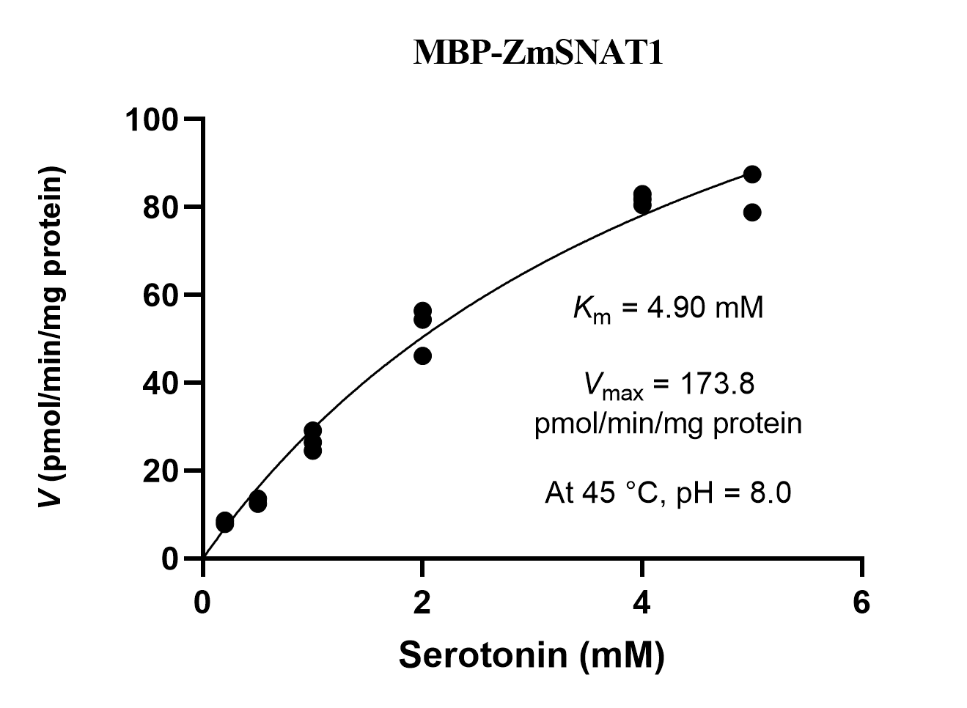


**Figure S1** Kinetic study of the recombinant MBP-ZmSNAT1 protein. The reaction was performed in PBS buffer (pH = 8.0) with 0.5 mmol/L acetyl-CoA and a series of concentrations of serotonin for 1 hour at 45 ℃. The *Km* and the *Vmax* were determined using Michaelis-Menten kinetics.

**Figure S2**


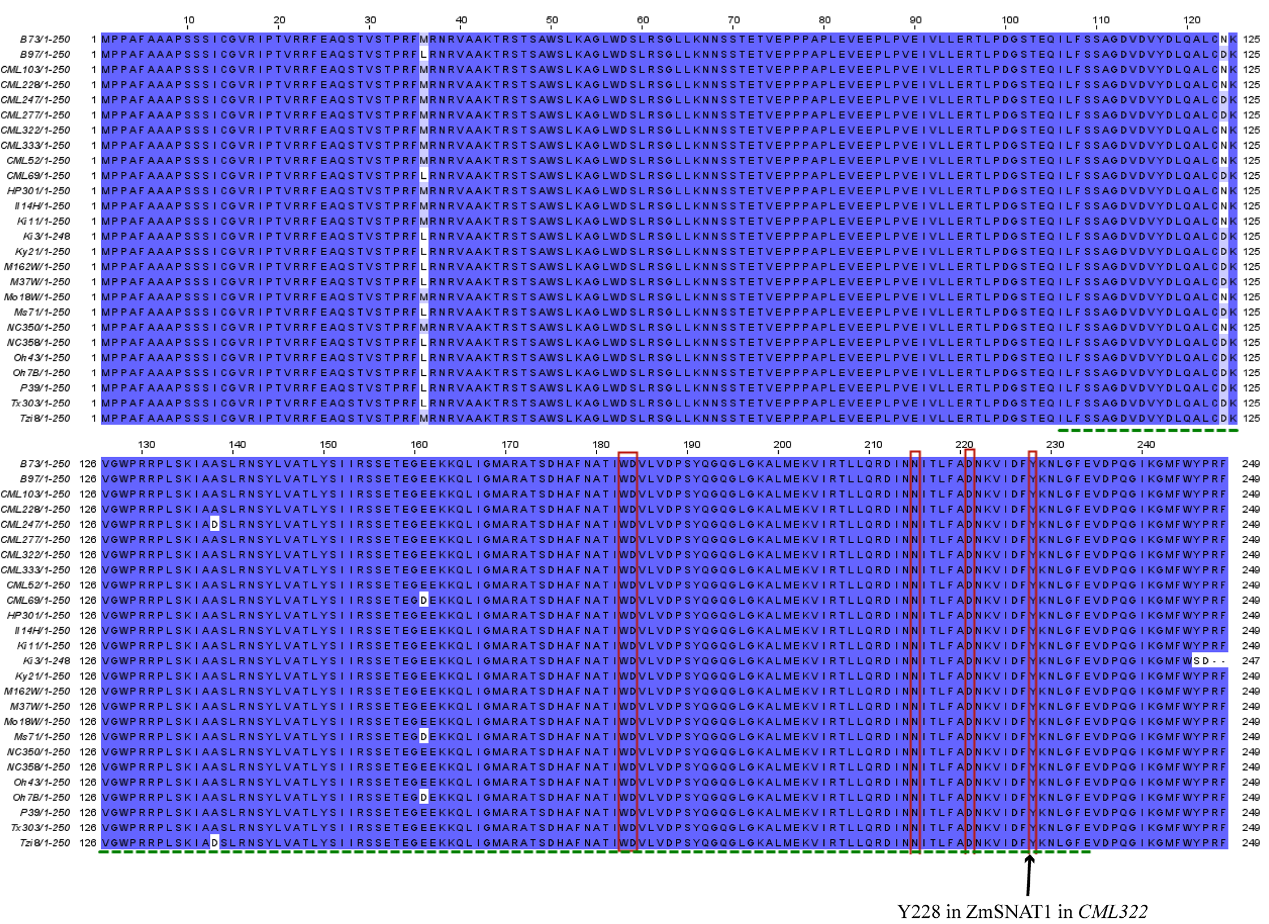


**Figure S2** The alignment of ZmSNAT1 amino acid sequences in 26 maize varieties.

The green dotted line represents the structural domain and the red box represents the amino acid sites that may bind to serotonin.

**Figure S3**

**
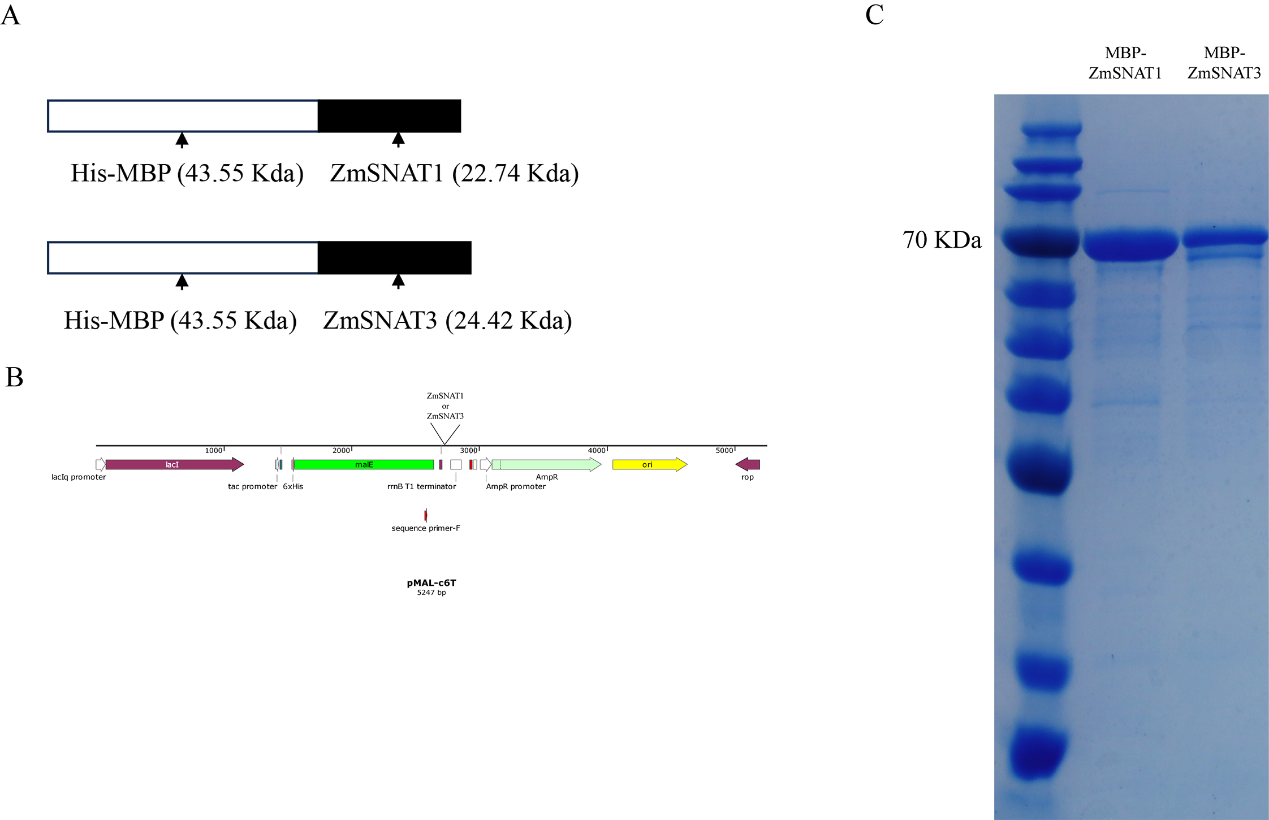
**

**Figure S3** Expression and purification of MBP-ZmSNATs recombinant proteins. **(A)** Construction of ZmSNAT expression vectors; **(B)** Schematic diagram of the composition of recombinant proteins; **(C)** Affinity purification of the recombinant ZmSNAT1 and ZmSNAT3 from *E.coli*. Protein samples were separated on 12% (w/v) SDS-PAGE gels and stained with Coomassie blue.

**Table S1** The primers used in this study for constructing the vector and quantitative real-time PCR.

| Primers | Sequence (5’→3) |
| --- | --- |
| ZmSNAT1-MBP-F | ggggagaacctgtacttccagtctACATCAGCTTGGTCCTTGAAG |
| ZmSNAT1-MBP-R | gatatcgcggccgcccatcagcatCTAAAATCTGGGGTACCAGAACATG |
| ZmSNAT3-MBP-F | ggggagaacctgtacttccagtctGCCACGGAGGCGGGGGTC |
| ZmSNAT3-MBP-R | gatatcgcggccgcccatcagcatTCATTGATCATCGTGAACCGG |
| ZmSNAT1-HIS-F  ZmSNAT1-HIS-R  ZmSNAT3-HIS-F  ZmSNAT3-HIS-R | caccaccatcacgtgggtaccACATCAGCTTGGTCCTTGAAG  gtcatcattcgaaccggtaccCTAAAATCTGGGGTACCAGAACATG  caccaccatcacgtgggtaccGCCACGGAGGCGGGGGTC  gtcatcattcgaaccggtaccTCATTGATCATCGTGAACCGG |
| qPCR-*ZmSNAT1*-MBP-F | TCCTTGAAGGCTGGCTTGTG |
| qPCR-*ZmSNAT1*-MBP-R  qPCR-*ZmSNAT3*-MBP-F  qPCR-*ZmSNAT3*-MBP-R  qPCR-*ZmTUB*-F  qPCR-*ZmTUB*-R | ACATCAACATCTCCGGCTGAA  CAAGAGGGGGCTTCTTGCG  ATCTCCTCCCCAACCCGTG  CTACCTCACGGCATCTGCTATGT  GTCACACACACTCGACTTCACG |

Note: Lowercase letters are homology arms.
